# Nursing care management strategies to address the COVID-19 pandemic

**DOI:** 10.1590/0034-7167-20230254

**Published:** 2025-01-10

**Authors:** Gabriela Marcellino de Melo Lanzoni, Leonardo Pereira de Sousa, Viviane Pecini da Cunha, Ana Lúcia Schaefer Ferreira de Mello, Alexandre Pazetto Balsanelli, José Luís Guedes dos Santos

**Affiliations:** I Universidade Federal de Santa Catarina. Florianópolis, Santa Catarina, Brazil; II Universidade Federal de São Paulo. São Paulo, São Paulo, Brazil

**Keywords:** Nursing Care, Health Management, COVID-19, Coronavirus Infections, Hospital Care, Atención de Enfermería, Gestión en Salud, COVID-19, Infecciones por Coronavirus, Atención Hospitalaria

## Abstract

**Objective:**

To characterize nursing care management strategies for addressing the COVID-19 pandemic.

**Method:**

A descriptive, qualitative study conducted with 22 nurse professionals at a University Hospital in Southern Brazil. Data collection through interviews in June and August 2021, analyzed according to Bardin’s Content Analysis and the theoretical framework of complex thinking.

**Results:**

The identified strategies were organized into four categories: Reorganization of health services; People management and emergency admission; Multiprofessional articulation; and Bedside nursing care.

**Final Considerations:**

Professional performance revealed a complex interplay between leadership and care management practices, even in the face of working condition restrictions, and were understood as crucial in the pandemic scenario.

## RESUMEN

**Objetivo:**

Caracterizar las estrategias de gestión del cuidado de enfermería para hacer frente a la pandemia de COVID-19.

**Método:**

Estudio descriptivo, cualitativo, realizado con 22 profesionales de enfermería en un Hospital Universitario en el Sur de Brasil. Recolección de datos a través de entrevistas en junio y agosto de 2021, analizados según el Análisis de Contenido de Bardin y el marco teórico del pensamiento complejo.

**Resultados:**

Las estrategias identificadas se organizaron en cuatro categorías: Reorganización de los servicios de salud; Gestión de personas y admisión de emergencia; Articulación multiprofesional; y Cuidado de enfermería en la cabecera.

**Consideraciones Finales:**

El desempeño profesional reveló una compleja interacción entre el liderazgo y las prácticas de gestión del cuidado, incluso ante las restricciones de las condiciones laborales, y se entendieron como cruciales en el escenario pandémico.

## INTRODUCTION

The COVID-19 pandemic has been a public health emergency of international importance^(1)^. In Brazil, a variety of actions were implemented to prevent and control the disease, aiming to contain and mitigate its spread^(2)^. Within the healthcare sector, hospital units underwent structural changes and adjustments in their work processes to ensure optimal patient care outcomes and to preserve the physical and psychological well-being of frontline teams^(3)^.

Among these teams, nursing is acknowledged as the critical core in combating COVID-19, playing an essential role in patient care^(3)^. Therefore, in the nursing workflow, there is a nexus between management and nursing care dimensions^(4)^. Nursing care management is conceptualized as the coordination and integration of caregiving and managerial activities, facilitated through leadership, as well as interactive, communicative, and cooperative relationships, orchestrated by the nurse with the nursing team, other health professionals, and patients^(5)^. However, the pandemic’s overarching environment has obscured the visibility of these work processes, which are marked by low wages, long hours, suffering, and death^(6,7)^, compounding the existing complexities and presenting even greater challenges for nurse managers and leaders during this time^(8)^.

In their managerial roles, nurses devise strategies to organize work and human resources, aiming to provide the team with the conditions necessary for effective patient care. Moreover, the care dimension specifies the health care needs with a focus on nursing interventions^(4)^. This systemic and complex thinking in nursing is characterized by a holistic approach to the patient and their families, recognizing the interdependence of diverse knowledge bases, services, and health sectors^(8)^. This approach was particularly evident amidst the chaos caused by shortages of supplies and healthcare personnel^(9)^, requiring innovative actions, creativity, and resilience during the pandemic.

Confronted with the sanitary and humanitarian crisis triggered by the COVID-19 pandemic, and the necessity to comprehend the multiple interactions involving nursing actions in both prevention and treatment of affected patients—which has led to suffering and even death among healthcare workers—the following question has been posed: What care management strategies have nurses employed to address the COVID-19 pandemic in hospital settings?

## OBJECTIVE

To characterize the nursing care management strategies utilized to address the COVID-19 pandemic at a university hospital in southern Brazil.

## METHOD

### Ethical Considerations

This research was approved by the Research Ethics Committee and conducted in accordance with Resolution No. 466/2012 of the National Health Council. All participants signed an Informed Consent Form.

### Study Type

This qualitative study is exploratory-descriptive. It was developed following the checklist criteria from the Consolidated Criteria for Reporting Qualitative Research (COREQ)^(10)^.

### Study Setting

The study was carried out at a University Hospital in the southern region of Brazil. Classified as a large general hospital, it accommodates 216 beds. The selected units for participant recruitment included the surgical clinic, medical clinic, adult COVID Intensive Care Unit (ICU), and adult COVID Emergency department.

### Population or Sample

The sample comprised 22 nurses working at the study location who met the following inclusion criteria: being a registered nurse with at least three months of direct care experience with COVID-19 positive patients during the pandemic.

### Methodological Procedures

Participants were invited to participate in the study via email and/or personally by the lead researcher. Upon agreeing, participants read and signed the Informed Consent Form before participating in the interview. At the interview’s conclusion, participants were asked to suggest other nurses who matched the research profile. This facilitated the use of the snowball sampling technique to extend invitations and assemble a representative sample.

### Data Collection and Organization

Data was collected from June to August 2021 through individual semi-structured interviews that were audio-recorded. The interviews were conducted either in-person at the institution or online using WhatsApp® or Google Meet®, based on the participants’ preferences. Each interview lasted approximately 19 minutes, was transcribed verbatim, and the transcripts were returned to the participants for comments or corrections. The sample size was determined by reaching theoretical saturation of the data, in line with the study’s methodology and objectives.

### Data Analysis

Data analysis employed Laurence Bardin’s Content Analysis technique^(11)^. As a research technique, content analysis aims to interpret through an objective and systematic description of message content. The research included three phases: pre-analysis, material exploration, and information treatment, inference, and interpretation. Codes were organized in Microsoft Word, grouped by similarity of ideas, resulting in four categories and eight sub-categories that aligned with the study’s objectives.

The pre-analysis phase involved organizing, operationalizing, and systematizing initial ideas. Subsequently, documents were subjected to analysis, hypotheses and objectives were formulated, and indicators were developed to support the final interpretation. Material exploration entailed systematic text analysis through coding operations, decomposition, or enumeration. The final phase dealt with information treatment, inference, and interpretation, where categories served as units of analysis to illuminate the obtained information^(9)^.

## RESULTS

The majority of participants were female (77.2%), aged between 30 and 39 years (54.5%), held postgraduate specialization degrees (59.1%), and worked in the adult Intensive Care Unit (45.5%). Table 1 characterizes the participants in terms of sociodemographic data, educational background, and professional involvement.

**Table 1:** Characterization of Research Participants. Florianópolis, SC, Brazil, 2021

Characteristics	n	%
Gender		
Female	17	77.2
Male	05	22.8
Age		
20 to 29 years	02	9.1
30 to 39 years	12	54.5
40 to 49 years	07	31.8
50 to 59 years	01	4.6
Level of Education		
Undergraduate	01	4.5
Specialization (lato sensu)	13	59.1
Master's degree	08	36.4
Work Unit		
Adult Intensive Care Unit	10	45.5
Adult Medical Clinic	07	31.8
Adult Emergency	05	22.7
Time Working in the Unit		
Less than 1 year	07	31.9
1 to 5 years	11	50.0
6 to 10 years	04	18.1
Professional Experience		
Less than 1 year	01	4.6
5 to 10 years	06	27.2
11 to 15 years	07	31.9
16 to 20 years	03	13.6
21 to 25 years	05	22.7
Time Working at the Institution		
Less than 1 year	07	31.9
1 to 5 years	11	50.0
6 to 10 years	04	18.1
Time Working with COVID-19 Patients		
3 to 7 months	10	45.4
8 to 12 months	01	4.6
1 to 2 years	11	50
Weekly Work Hours		
30 hours	06	27.3
36 hours	15	68.1
40 hours	01	4.6
Work Shift		
Daytime	10	45.5
Morning	04	18.2
Night	08	36.3
Type of Employment Contract		
Consolidation of Labor Laws, Brazil	17	77.2
Public Service Exam	05	22.8
Second Job?		
Ye s	06	27.3
No	16	72.7
TOTAL	22	100%

### Results organization

Consequently, the results were organized into four categories, corresponding to the synthesis of the significances derived from the data analysis of the study. These categories are: Reorganization of Health Services, People Management and Emergency Admissions, Multiprofessional Coordination, and Bedside Nursing Care (Chart 1).

**Chart 1:** Categories and Subcategories Emerging from the Research. Florianópolis, SC, Brazil, 2021

Categories	Subcategories
Reorganization of Health Services	Modifications in Units and Flows Rationing of Personal Protective Equipment and Hospital Supplies
People Management and Emergency Admissions	Increased Responsibility for More Experienced Workers Exercise of Leadership
Multiprofessional Coordination	Use of Protocols In-service Education
Bedside Nursing Care	Prioritization of Essential Care Support in Activities of Other Care Areas

### Reorganization of Health Services

This category includes two subcategories. The “Modifications in Units and Flows” subcategory describes changes implemented to meet emerging needs. Nurses actively contribute by making suggestions, indicating possibilities, and offering solutions, particularly regarding the flow of people within the institution and the areas designated for the health team to don protective gear. However, they have observed weaknesses in organizational support, often leading to feelings of abandonment and the verticalization of work relationships. This situation, conducive to the fragmentation of care, was fraught with uncertainties, communication problems, and managerial challenges.

*We didn’t know what we were going to face. In terms of services, some units closed, others opened, so the unit I belonged to was closed, and people were relocated.* (E2)

*The word is ‘challenge.’ From the beginning, we had to adapt. We had one ICU; now we have two, a COVID-19 ICU and a General ICU. It has been very difficult from the start, with coordination proving challenging due to issues with materials and human resources.* (E1)

Management of material resources also proved challenging at the institution. The subcategory “Rationing of Personal Protective Equipment and Hospital Supplies” highlights the primary strategy for dealing with supply shortages that impacted certain care units, leading to problems ranging from direct care to the management of internal departments, as demonstrated:

*There was a significant issue—the scarcity of materials; we even ran out here at our hospital.* [...] *Rationing was the only possible way forward.* (E18)

*There was a considerable shortage of materials in the service; we couldn’t do without, so we improvised. We divided materials among ourselves so that not everyone had to use all the PPE and run out by the end of the shift.* (E9)

### People Management and Emergency Admissions

This category is underpinned by two subcategories. The first, “Increased Responsibility for More Experienced Workers,” describes a scenario characterized by an abrupt and massive demand for beds and a high workload for nurses, accompanied by the emergency admission of new healthcare workers. Confronted with the professional inexperience of these new hires and the need to align with hospital routines, the implemented strategy recognized the more experienced workers who were better adapted to the new context. This situation highlighted the physical and mental burden on these professionals as they supervised new employees amidst a highly tense care dynamic, due to the complexity and severity of the patients, as reflected in the following statements:

*We faced some difficulties because many newly graduated professionals were admitted through an emergency selection process. We were in the middle of a pandemic, amidst chaos, and then you receive new colleagues who still need training, so that part was challenging.* (E10)

*The most critical cases were assigned to the most experienced, the less critical to the newcomers, and we were constantly teaching. We managed 20 beds with only three nurses, of whom only one had more experience; there were many new technicians and nurses, and we had colleagues who couldn’t withstand this pressure.* (E17)

The second subcategory, “Exercise of Leadership,” highlights how nurses managed to overcome fear and direct the team amid adversity. The need to devise new processes in response to various challenges encountered in healthcare during the pandemic became a complex task that provided new experiences and numerous lessons. Occasionally, nurses felt limited and insecure, mainly due to the reactions of other workers; nevertheless, they enacted their leadership practices, occasionally supported by organizational backing.

Routinely, the insufficient staffing of nursing personnel affected care units alarmingly as the patient numbers increased and the workers became overwhelmed. Nurses noted that the shortage of professionals compromised the ability to provide risk-free care. In this context, participants indicated that the leadership of nurses was crucial in guiding overloaded, insecure, and demotivated teams, acting with purpose and feeling supported by peers in a challenging work environment, as noted in the statement:

*In terms of leadership, colleagues did not complain; we managed to perform all services. I managed to execute the nursing process, you know? And the predominant feelings were fear and insecurity. However, the leadership exerted by the nurse, and by myself, helped me overcome that fear.* (E16)

*I had to heavily focus on leadership to ensure the service flowed smoothly, because we lacked support and there was considerable frustration, yet at the same time, the patients needed us. Thus, we had to work extensively with the team, not only with the nursing technicians but also with the medical colleagues, showing them that we needed their cooperation as well.* (E11)

### Multiprofessional Coordination

Training and professional development significantly enhanced the quality of nursing care. For these workers, a continuous search for literature and engagement in training practices provided greater security in the development of care practices, especially at the onset of the pandemic.

The subcategory “Use of Protocols” underscores a strategy that was evident in the dynamics of nursing and multiprofessional support. This collaboration led to improved synchronization across various professions and a more robust flow of care amid high demand and patient turnover, as illustrated in the following statements:

*Many SOPs and protocols were developed to ensure proper execution. This ensured that our collaborative work was successful and continually improved.* (E5)

*The multidisciplinary team functioned smoothly. We encountered few problems. Tasks were divided, and everyone played their part. The physiotherapist, nutritionist, speech therapist, and doctor—it was all well-organized. Everything was centered on the patient, aiming to provide better care.* (E15)

The subcategory “Health Education” enhanced and broadened the understanding of care provided by the members of the nursing and multiprofessional teams. The collaborative efforts among various professionals facilitated the establishment of a comprehensive care network, offering diverse approaches to specific issues and enabling the selection of the most suitable therapies for patients, as noted in the following comments:

*Training sessions and simulations were conducted. I noticed that people felt more secure afterward.* (E6)

*Before the onset of COVID-19, there were preparatory courses, including online courses; the teams prepared themselves and developed protocols.* (E14)

### Bedside Nursing Care

The increase in the number of severely ill patients admitted was not accompanied by the immediate hiring of additional professionals, resulting in a chaotic scenario. In this context, the subcategory “Prioritization of Essential Care” represented a practical strategy, focusing on measures to sustain the lives of COVID-19 patients. It was noted that some nursing process records were not completed to prioritize direct bedside care, as mentioned by the participants:

*In the emergency room, we managed bed flow by completing systematization with history, progression, and prescription. For patients on stretchers, we manually updated each shift, maintaining this practice throughout the pandemic.* (E16)

*I had a shift with three technicians and me as the nurse, managing three intubated and 25 patients in total, creating a war-like scenario.* (E2)

Furthermore, with the virus’s high transmissibility rate, adjustments were made in unit flows. The reorganization of care practices significantly impacted nursing, as certain workers, including laboratory support, hospitality, and nutrition services, did not access contaminated units. As a result, the subcategory “Assistance in Activities of Other Care Areas” emerged as a strategy by the nursing team to ensure the fulfillment of patients’ basic human needs. However, this approach increased virus exposure and workload, leading to compromised care and dissatisfaction among nursing staff, as illustrated by the following statements:

*The nursing team assumed the role of the laboratory; we collected all blood tests because the lab team was restricted from entering to avoid increasing the flow of potentially contaminating professionals in the hospital. For food delivery, the dietary aide would leave it at the emergency room door, and the nurse would then pick it up and distribute it to each patient, a measure taken to reduce the infection rate within the hospital.* (E9)

*Essentially, the nursing team performed all roles, from transporting for exams to delivering food and, after the speech therapist’s assessment, also offering assisted diets.* (E2)

Therefore, the importance of nursing care must be acknowledged. In this regard, it was observed that care practices focused on basic needs were implemented, as demonstrated:

*We provided comprehensive care to the patient. We managed to better control our tasks thanks to a well-organized assignment and scale system. This setup ensured that some nurses focused solely on resuscitation, others on caring for intensive patients, some on hygiene, and others on medication management, resulting in more humanized and risk-free care.* (E11)

*We always prioritize patients who are more vulnerable, paying extra attention to them, remaining vigilant, and maintaining humanization, especially since these patients cannot receive visitors.* (E9)

## DISCUSSION

Developing strategies for care management involves complex activities and numerous relationships within the workplace. From this perspective, the present study highlighted the care management strategies employed during the pandemic, despite adversities, in hospital units. These strategies included the reorganization of health services and workflows, rationing of Personal Protective Equipment (PPE) and hospital supplies, as well as people management, emphasizing the leadership of nurses and the expansion of responsibilities assigned to more experienced workers with the emergency admission of nursing staff.

An integrative review comparing the work processes of Brazilian nurses with those from other countries across various continents revealed that in fulfilling their duties within health institutions—coordinating team members’ work processes, guiding the work processes of other health workers to ensure service delivery, structuring the environment, and performing technical care procedures—nurses consistently consider the needs of patients using these health services^(12)^. Consequently, the nurses in this study highlighted the challenges brought about by the lack of supplies, which affected everything from direct care to the management of care itself. This issue was not an isolated incident but rather part of the broader global context^(13,14)^, as also shown in studies from the United States^(15)^ and China^(16)^.

Faced with the pandemic, various adjustments necessitated the use of care protocols and training alongside the multiprofessional team. Despite numerous changes, nursing continued to be responsible for care coordination, prioritizing direct patient care, and taking on activities from other care areas, even though it was overloaded. The staffing of nursing personnel illustrated a reality commonly seen in health institutions; however, regarding care management and the exercise of leadership by nurses, they demonstrated the effectiveness of the leadership role in organizing care and in the emergency hiring of personnel. Supporting the findings of this research, despite challenges, the professional practice environment for nurses was rated as favorable both before and after the pandemic in a São Paulo hospital^(17)^, considering the suite of interventions and the management of available resources. It is crucial to note that the unfavorable assessment of team adequacy, along with personnel discrepancies and workload overload, are recognized as common problems in many countries and are particularly alarming in Brazilian public hospitals^(18,19)^.

As demonstrated in this study, the healthcare needs prompted by the pandemic have far exceeded the capacities of hospitals and health systems globally, creating a stressful environment for frontline nursing staff^(20)^. This stress arises because providing care involves the contingent management of processes and material resources, which, in the context of the pandemic, proved significant and challenging. It necessitated the establishment of field hospitals to support certain municipalities nationally^(21)^. Thus, chaotic scenarios can serve as catalysts for change, facilitating the identification of new solutions or reorganizations for effective problem management^(22)^.

Nurses on the frontline required substantial organizational support, both structural and emotional, to help them address emerging challenges and ensure the safety of both the workers and their patients^(23)^. Inadequate staffing levels have led to restricted care, high workloads, and increased conflicts. Consequently, a study grounded in international scientific evidence recommends forming skilled teams with an adequate number of professionals and strategically limiting their direct contact with confirmed or suspected cases. Dedicated teams should exclusively manage this population to minimize the risk of virus transmission^(24)^.

Exercising leadership during this pandemic underscores the importance of autonomy, decision-making capability, and directing ethical and competent care practices, as highlighted by the findings of this study. It is crucial to acknowledge the complex nature of human beings and the contexts in which they exist, treating individuals as entities with diverse characteristics that must be considered within their relationships and interrelations^(25)^. Therefore, focusing on a care model that caters to the specific needs of patients, prioritizes their concerns, and utilizes all available resources underscores a deep understanding of the interdependence of care practices^(7)^.

Hence, the role of nurses during the pandemic necessitated intricate coordination between leadership and advanced care management practices to safeguard physical safety and lessen the emotional burdens on the nursing team, while striving to deliver the best possible healthcare within the constraints of current working conditions^(13,14,26)^.

In light of the importance of qualified and specialized nursing care, despite limitations in material and human resources, participants described a routine based on simulations and continuous professional updates in hospital units. There is a strong consensus in international literature that simulation activities are effective strategies frequently used to help healthcare professionals achieve high levels of competence and provide safer care^(27,28)^. Furthermore, these simulations and professional updates are viewed as beneficial strategies for ongoing health education, enhancing meaningful learning, particularly with the emphasis on digital education technologies^(29,30)^.

Consequently, the complexity introduces a method of integrating knowledge from multiple fields: a transdisciplinary approach that invents, imagines, reflects, and analyzes everyday situations, offering solutions^(31)^. Therefore, structuring actions and involving all nursing professionals and the multiprofessional team is crucial for making decisions that gain broader consensus and can be implemented more swiftly and effectively. This includes the creation of multiprofessional Crisis Management Committees^(32)^ and the initiation of multidisciplinary rounds^(33)^ in hospitals in the southeast and south of Brazil, respectively. Thus, jointly establishing care parameters that can achieve a high degree of efficiency is essential^(32,33)^.

Even in a chaotic scenario, taking on the roles of other professionals and without support staff, nurses and nursing technicians fought for lives and maintained dignified and qualified professional practice^(34)^. In this context, there was active involvement in the processes of coordination, professional and material management, and actions related to care protocols across various levels of complexity in other regions of Brazil^(35,36)^, supporting the findings of this study.

Bedside nursing promotes improvements in the quality of care through the application of the nursing process, which includes organized and systematized care planning^(35)^. Additionally, this bedside care model seeks to reintegrate the nurse into the locus of direct, specialized care, underpinned by highly specialized professional training^(37)^.

### Study Limitations

This study presents several significant limitations. First, it is important to note that the research was conducted solely at one university hospital in Southern Brazil, potentially limiting the applicability of the results to other healthcare institutions or geographic regions. Additionally, due to the specificities associated with the care of critically ill patients, integrating and consolidating data from different hospitalization areas may be viewed as a limitation of the study.

### Contributions to the Field of Nursing

This study offers valuable insights into nursing management and its implications for community health, providing a basis for discussion in educational and healthcare institutions. It highlights crucial findings that help the public understand the managerial role of nursing and its impact on population health, as well as fosters discussions on the topic in educational and healthcare settings through the sharing of management experiences. The potential for nursing in team leadership and project management during crises is believed to be enhanced with institutional investments aimed at improving post-pandemic internal processes.

## References

[1] World Health Organization (WHO (2020). WHO declares Public Health Emergency on novel coronavirus [Internet].

[2] Cavalcante JR, Cardoso-dos-Santos AC, Bremm JM, Lobo A P, Macário EM, Oliveira WK (2020). COVID-19 in Brazil: evolution of the epidemic up until epidemiological week 20 of 2020.. Epidemiol Serv Saúde..

[3] Araujo PMCG, Bohomol E, Teixeira TAB (2020). Nursing management in a public general hospital accredited in facing the COVID-19 pandemic.. Enferm Foco [Internet].

[4] Senna MH, Drago LC, Kirchner AR, Santos JLG, Erdmann AL, Andrade SR (2014). Meanings of care management built throughout nurses’ professional education.. Rev RENE..

[5] Mororó DDS, Enders BC, Lira ALBC, Silva CMB, Menezes RMP (2017). Concept analysis of nursing care management in the hospital context.. Acta Paul Enferm..

[6] Araújo ST, Silva SH, Silva MN, Coelho ACC, Pires CGS, Melo CMM (2018). Job insecurity among nurses, nursing technicians and nursing aides in public hospitals.. Rev Esc Enferm USP..

[7] Santos JNMO, De La Longuiniere ACF, Vieira SNS, Amaral APS, Sanches GJC, Vilela ABA (2019). Occupational Stress: the Exposure of an Emergency Unit Nursing Team.. Rev Pesqui Cuid Fundam..

[8] Stein Backes D, Gomes RCC, Rupolo I, Büscher A, Silva MJP, Ferreira CLL (2022). Leadership in nursing and health in light of complex thinking.. Rev Esc Enferm USP..

[9] Santos JLG, Lanzoni GMM, Costa MFBNA, Debetio JO, Sousa L P, Santos LS (2020). How are university hospitals in Brazil facing the COVID-19 pandemic?. Acta Paul Enferm..

[10] Tong A, Sainsbury P, Craig J (2007). Consolidated criteria for reporting qualitative research (COREQ): a 32-item checklist for interviews and focus groups.. Int J Qual Health Care..

[11] Bardin L (2015). Content analysis.

[12] Leal JAL, Melo CMM (2018). The nurses’ work process in different countries: an integrative review.. Rev Bras Enferm..

[13] Silva KR, Souza FG, Roquete FF, Faria SMC, Peixoto BCF, Vieira A (2020). Allocation of resources for health care in COVID-19 pandemic times: integrative review.. Rev Bras Enferm..

[14] Sharp A, Jain V, Alimi Y, Bausch DG (2021). Policy and planning for large epidemics and pandemics: challenges and lessons learned from COVID-19.. Curr Opin Infect Dis..

[15] Emanuel EJ, Persad G, Upshur R, Thome B, Parker M, Glickman A (2020). Fair Allocation of Scarce Medical Resources in the Time of Covid-19.. N Engl J Med..

[16] Liu Y, Wang H, Chen J, Zhang X, Yue X, Ke J (2020). Emergency management of nursing human resources and supplies to respond to coronavirus disease 2019 epidemic.. Int J Nurs Sci..

[17] Danno CH, Bohomol E, Gasparino RC (2022). Nursing practice environment before and during the COVID-19 pandemic.. Acta Paul Enferm..

[18] Branco LLWV, Beleza LO, Luna AA (2017). Nursing workload in neonatal ICU: application of the Nursing Activities Score.. Rev Pesqui Cuid Fundam..

[19] Rodrigues CM, Costa KES, Antunes AV, Gomes FA, Rezende GJ, Silva DV (2017). Workload and staffing of nursing personnel in Intensive Care Units.. Rev Aten Saúde..

[20] Timmis K, Brüssow H (2020). The COVID-19 pandemic: some lessons learned about crisis preparedness and management, and the need for international benchmarking to reduce deficits.. Environ Microbiol..

[21] Dzivielevski AMO, Costa ADMB, Paraizo-Horvath CMS, Silva SA, Sanches RS, Resck ZMR (2021). Structuring a field hospital in the COVID-19 pandemic: experience report.. Saúde Colet..

[22] Ceylan Z (2020). Estimation of COVID-19 prevalence in Italy, Spain, and France.. Sci Total Environ..

[23] Labrague LJ, De Los Santos JAA (2020). COVID-19 anxiety among front-line nurses: predictive role of organisational support, personal resilience and social support.. J Nurs Manag..

[24] Ferioli M, Cisternino C, Leo V, Pisani L, Palange P, Nava S (2020). Protecting healthcare workers from SARS-CoV-2 infection: practical indications.. Eur Respir Rev..

[25] Morin E (2011). Introduction to Complex Thinking.

[26] Cassiani SHB, Lira Neto JCG (2018). Nursing Perspectives and the “Nursing Now” Campaign.. Rev Bras Enferm..

[27] Lamberta, M, Aghera A (2020). Latent safety threat identification via medical simulation.

[28] Goolsarran N, Hamo CE, Lane S, Frawley S, Lu WH (2018). Effectiveness of an interprofessional patient safety team-based learning simulation experience on healthcare professional trainees.. BMC Med Educ..

[29] Knobel A, Overheu D, Gruessing M, Juergensen I, Struewer J (2018). Regular, in-situ, team-based training in trauma resuscitation with video debriefing enhances confidence and clinical efficiency.. BMC Med Educ..

[30] Malfussi LBH, Nascimento ERP, Baptista RCN, Lazzari DD, Martini JG, Hermida PMV (2021). In situ simulation in the permanent education of the intensive care nursing team.. Texto Contexto Enferm..

[31] Morin E (2002). Education and complexity: the seven knowledges and other essays.

[32] Laselva CR (2020). Technical and managerial actions of nursing at Hospital Israelita Albert Einstein to meet during the COVID-19 pandemic.. Enferm Foco..

[33] Guzinski C, Nogueira A, Lopes M, Flor J, Migliavaca J, Tortato C (2019). Best practices for effective communication: the experience of interdisciplinary rounds in orthopedic surgery.. Rev Gaúcha Enferm..

[34] Martins ALX, Crisostomo Júnior VJL, David HMSL (2021). Social control and nursing performance in defense of life in the COVID-19 pandemic.. Rev Bras Enferm..

[35] Sousa AR, Santos GLA, Da Silva RS, Carvalho ESS (2020). Reflections on the Nursing Process in the work of nurses facing the COVID-19 pandemic.. Enferm Foco..

[36] Bitencourt JVOV, Meschial WC, Frizon G, Biffi P, Souza JB, Maestri E (2020). Nurse’s protagonism in structuring and managing a specific unit for Covid-19.. Texto Contexto Enferm..

[37] Rezende LC, Vilela GS, Caram CS, Caçador BS, Brito MJM (2021). Bedside nurses’ care model: challenges and perspectives for an innovative practice.. Rev Gaúcha Enferm..

