# Supplementary material for: Nursing care management strategies to address the COVID-19 pandemic
Source: Rev Bras Enferm. 2025 Jan 10;77(Suppl 1):e20230254. doi: 10.1590/0034-7167-20230254 (PMC11726817; doi:10.1590/0034-7167-20230254)
Supplement: Translation

# Translation: Estratégias de gestão do cuidado de enfermagem para enfrentamento da pandemia de COVID-19

**DOI:** 10.1590/0034-7167-20230254pt

**Authors:** Gabriela Marcellino de Melo Lanzoni, Leonardo Pereira de Sousa, Viviane Pecini da Cunha, Ana Lúcia Schaefer Ferreira de Mello, Alexandre Pazetto Balsanelli, José Luís Guedes dos Santos

**Affiliations:** I Universidade Federal de Santa Catarina. Florianópolis, Santa Catarina, Brasil; II Universidade Federal de São Paulo. São Paulo, São Paulo, Brasil

**Keywords:** Cuidados de Enfermagem, Gestão em Saúde, COVID-19, Infecções por Coronavirus, Assistência Hospitalar

## Abstract

**Objetivo:**

Caracterizar as estratégias de gestão do cuidado de enfermagem para o enfrentamento da pandemia de COVID-19.

**Método:**

Estudo descritivo, qualitativo, realizado com 22 profissionais enfermeiros em um Hospital Universitário no Sul do Brasil. Coleta de dados por meio de entrevista nos meses de junho e agosto de 2021, analisados segundo a Análise de Conteúdo de Bardin e o referencial teórico do pensamento complexo.

**Resultados:**

As estratégias identificadas foram organizadas em quatro categorias: Reorganização dos serviços de saúde; Gestão de pessoas e admissão emergencial; Articulação multiprofissional; e Cuidado de enfermagem à beira-leito.

**Considerações Finais:**

A atuação profissional evidenciou uma complexa articulação entre liderança e práticas de gestão do cuidado, mesmo diante das restrições de condições de trabalho, e foram compreendidas como fundamentais diante do cenário pandêmico.

## INTRODUÇÃO

A pandemia da COVID- 19 representou uma emergência em saúde pública de importância internacional^(1)^. No Brasil, diferentes ações foram implementadas para prevenção e controle com o objetivo de conter e mitigar o avanço da doença^(2)^. No âmbito dos serviços de saúde, as unidades hospitalares passaram por modificações estruturais e em seus processos de trabalho para proporcionar os melhores resultados no atendimento ao paciente e para a preservação física e psicológica das equipes que estavam na linha de frente^(3)^.

Dentre as equipes atuantes, a enfermagem é reconhecida como o núcleo essencial na atuação contra a COVID-19, e desempenha um papel fundamental no cuidado^(3)^. Assim, no processo de trabalho do enfermeiro há uma conexão entre as dimensões de gestão e de cuidado de enfermagem^(4)^. A gestão do cuidado de enfermagem é conceituada como a articulação e integração entre as atividades cuidativas e gerenciais, por intermédio do exercício de liderança, relações interativas, comunicativas e cooperativas apropriadas pelo enfermeiro para com a equipe de enfermagem, demais profissionais de saúde e usuários^(5)^. No entanto, o macroambiente da pandemia limitou a visibilidade do processo de trabalho que tem sido caracterizado por baixos salários, longas jornadas de trabalho, sofrimento e morte^(6,7)^, tornando o que já era complexo, um desafio ainda maior para enfermeiros gestores e líderes durante a pandemia^(8)^.

O enfermeiro, ao atuar na gestão, formula ações voltadas para a organização do trabalho e dos recursos humanos, cujo objetivo é proporcionar condições adequadas para a equipe realizar a assistência ao paciente. E, a dimensão assistencial define as necessidades de cuidados em saúde com foco na intervenção do cuidado de enfermagem^(4)^. Assim, o pensamento sistêmico-complexo impresso no trabalho da enfermagem é caracterizado pela abordagem integral ao paciente e suas famílias, reconhecendo a interdependência dos diferentes saberes, serviços e setores de saúde^(8)^, amplamente verificado diante do caos criado pela escassez de insumos e de trabalhadores de saúde^(9)^, exigindo novas formas de atuar, criatividade e resiliência durante a pandemia.

Deste modo, diante da crise sanitária e humanitária gerada pela pandemia da COVID-19 e a necessidade compreender as múltiplas interações que englobam as diversas ações da enfermagem na prevenção e tratamento dos pacientes acometidos por essa doença, a qual ocasionou sofrimento ou mesmo mortes desses trabalhadores, buscou-se responder ao seguinte questionamento: Quais estratégias de gestão do cuidado foram utilizadas por enfermeiros para enfrentamento à pandemia de COVID-19 em ambiente hospitalar?

## OBJETIVO

Caracterizar as estratégias de gestão do cuidado de enfermagem para enfrentamento da pandemia de COVID-19 em um hospital universitário ao sul do Brasil.

## MÉTODO

### Aspectos éticos

Esta pesquisa foi aprovada pelo Comitê de Ética em Pesquisas, sendo desenvolvida de acordo com a Resolução nº 466/2012, do Conselho Nacional de Saúde. Na ocasião, os participantes assinaram o Termo de Consentimento Livre e Esclarecido.

### Tipo de estudo

Trata-se de um estudo do tipo exploratório-descritivo, de natureza qualitativa. Para seu desenvolvimento, foram seguidos os critérios da lista de verificação do *Consolidated criteria for reporting qualitative research* (COREQ) ^(10)^.

### Cenário do estudo

Realizado em um Hospital Universitário, localizado no Sul do Brasil. Este hospital é classificado como geral e de grande porte, conta com 216 leitos. Considerando o perfil de participantes, foram selecionadas as unidades de clínica cirúrgica, clínica médica, Unidade de Terapia Intensiva (UTI) adulto COVID; e, Emergência adulto COVID.

### População ou amostra

A amostra foi constituída por 22 enfermeiras que trabalhavam no local do estudo e que obedeceram aos seguintes critérios de inclusão: ser enfermeiro e ter experiência na assistência direta aos pacientes positivados para COVID-19 durante a pandemia por, no mínimo, três meses.

### Procedimentos metodológicos

Os participantes foram convidados a fazer parte do estudo por meio de e-mail convidativo e/ou pessoalmente pelo pesquisador responsável. Mediante o aceite, o termo de consentimento livre e esclarecido foi lido e assinado, para então ser realizada a entrevista. Ao final da entrevista foi solicitado ao participante indicar outros enfermeiros que atendiam ao perfil de interesse da pesquisa. Assim, a técnica bola de neve foi utilizada para condução de novos convites e compor uma amostra representativa.

### Coleta e organização dos dados

A coleta de dados ocorreu entre os meses de junho e agosto de 2021, por meio de entrevista individual, semiestruturada, e audio-gravada, de forma presencial na própria instituição ou online com o uso da plataforma Whatsapp**®** ou Google Meet**®**, de acordo com a preferência do participante. Os questionamentos versavam sobre a sistematização do cuidado de enfermagem e sua rotina como enfermeiro na prestação de cuidados às pessoas com COVID-19, quais tecnologias, limites, dificuldades e potencialidades na realização dos cuidados. As entrevistas tiveram uma duração média de 19 minutos, todas foram transcritas na íntegra e devolvidas aos participantes para comentários e/ou correções. O tamanho da amostra foi determinado pela saturação teórica dos dados, de acordo com a metodologia e o alcance do objetivo do estudo.

### Análise dos dados

Para a análise dos dados, foi utilizada a técnica da Análise de Conteúdo, de Laurence Bardin^(11)^. A análise de conteúdo como técnica de investigação tem por finalidade a interpretação através de uma descrição objetiva e sistemática do conteúdo das mensagens. A pesquisa foi compreendida três fases: pré-análise, exploração do material e tratamento das informações, a inferência e a interpretação.

Os códigos foram organizados em editor de texto *Microsoft Word*, os quais foram agrupados por similaridade de ideias, gerando, a partir dos dados, quatro categorias e oito subcategorias que respondem o objetivo de estudo. Na pré-análise ocorreu a organização, opera-cionalização e a sistematização das ideias iniciais, posteriormente os documentos foram submetidos à análise, formulação das hipóteses e objetivos, e a elaboração de indicadores que respaldassem a interpretação final. A exploração do material consistiu na análise sistemática do texto através de operações de codificação, decomposição ou enumeração e a última fase compreendeu o tratamento das informações, inferência e a interpretação, onde as categorias utilizadas como unidades de análise ressaltam as informações obtidas ^(9)^.

## RESULTADOS

Dos participantes, a maioria era do sexo feminino (77,2%), idade entre 30 e 39 anos (54,5%), com nível de formação do tipo especialização *lato sensu* (59,1%) e como setor de atuação a Unidade de terapia intensiva adulto (45,5%). A Tabela 1 apresenta a caracterização dos participantes em questões sociodemográficas, de formação e de inserção profissional.

**Tabela 1:** Caracterização dos participantes da pesquisa. Florianópolis, SC, Brasil, 2021

Características	n	%
Sexo		
Feminino	17	77,2
Masculino	05	22,8
Idade		
20 a 29 anos	02	9,1
30 a 39 anos	12	54,5
40 a 49 anos	07	31,8
50 a 59 anos	01	4,6
Nível de formação		
Graduação	01	4,5
Especialização *lato sensu*	13	59,1
Mestrado	08	36,4
Unidade de trabalho		
Unidade de Terapia Intensiva Adulto	10	45,5
Clínica Médica Adulto	07	31,8
Emergência Adulto	05	22,7
Tempo de atuação na unidade		
< 01 ano	07	31,9
01 a 05 anos	11	50,0
06 a 10 anos	04	18,1
Tempo de experiência profissional		
< 01 ano	01	4,6
05 a 10 anos	06	27,2
11 a 15 anos	07	31,9
16 a 20 anos	03	13,6
21 a 25 anos	05	22,7
Tempo de trabalho na instituição		
< 01 ano	07	31,9
01 a 05 anos	11	50,0
06 a 10 anos	04	18,1
Tempo de atuação com pacientes COVID-19		
03 a 07 meses	10	45,4
08 a 12 meses	01	4,6
01 a 02 anos	11	50
Carga horário semanal de trabalho		
30h	06	27,3
36h	15	68,1
40h	01	4,6
Turno de trabalho		
Diurno	10	45,5
Matutino	04	18,2
Noturno	08	36,3
Tipo de vínculo trabalhista		
CLT	17	77,2
Concurso Público	05	22,8
Segundo emprego		
Sim	06	27,3
Não	16	72,7
TOTAL	22	100%

Por conseguinte, os resultados foram organizados em quatro categorias, correspondentes à síntese do aparato das significações oriundas da análise dos dados do estudo, a saber: Reorganização dos serviços de saúde, Gestão de pessoas e admissão emergencial, Articulação multiprofissional e Cuidado de enfermagem beira-leito (Quadro 1).

**Quadro 1:** Categorias e subcategorias emergentes pesquisa. Florianópolis, SC, Brasil, 2021

CATEGORIAS	SUBCATEGORIAS
Reorganização dos serviços de saúde	- Modificação nas unidades e fluxos - Racionamento de Equipamento de Proteção Individual e insumos hospitalares
Gestão de pessoas e admissão emergencial	- Ampliação da responsabilidade dos trabalhadores mais experientes - Exercício da liderança
Articulação multiprofissional	- Uso de protocolos - Educação em serviço
Cuidado de enfermagem beira-leito	- Priorização dos cuidados essenciais - Auxílio em atividades de outras áreas assistenciais

### Reorganização dos serviços de saúde

A presente categoria é composta por duas subcategorias. Na subcategoria, “Modificações nas unidades e fluxos”, foram descritas as alterações implementadas para suprir as necessidades emergentes, os enfermeiros contribuem ativamente realizando sugestões, indicando possibilidades e soluções, especialmente em relação ao fluxo de pessoas na instituição e ambientes para paramentação da equipe de saúde. Entretanto, perceberam fragilidades no suporte organizacional, muitas vezes, acarretando sentimento de abandono e verticalização das relações de trabalho. Não obstante, esse cenário – propício para a fragmentação do cuidado – esteve permeado de incertezas, problemas de comunicação e dificuldades gerenciais.

*Não sabíamos o que iríamos enfrentar. Em relação aos serviços, fechamento de algumas unidades, aberturas de outras, então, a unidade que pertencia fechou e as pessoas foram realocadas.* (E2)

*A palavra é desafio. Porque desde o início, tivemos que nos adaptar. Tínhamos uma UTI, agora nós temos duas, uma UTI COVID 19 e uma UTI Geral. Desde o início tem sido muito difícil, coordenar é desafiador devido às questões de material e de recursos humanos.* (E1)

A gestão de recursos materiais também demonstrou ser um desafio na instituição. A subcategoria “Racionamento de EPIs e insumos hospitalares” aponta a principal estratégia diante da falta de insumos atingiu determinadas unidades assistenciais, acarretando problemas desde o cuidado direto até mesmo na gerência de setores internos, como demonstrado:

*Teve uma grande questão, a escassez de materiais, chegou a faltar aqui no nosso hospital.* [...] *Racionar foi o caminho possível.* (E18)

*Teve muita falta de materiais no serviço, a gente não podia ficar sem, então, improvisamos, fazíamos a divisão de materiais entre nós, para não ter que todo mundo utilizar todos os EPIs e acabar no final do plantão*. (E9)

### Gestão de pessoas e admissão emergencial

Esta categoria é sustentada por duas subcategorias. A primeira, “Ampliação da responsabilidade dos trabalhadores mais experientes”, descreve inicialmente o cenário de demanda abrupta e massiva dos leitos e a alta carga de trabalho dos enfermeiros, bem como a admissão emergencial de novos trabalhadores de saúde. Diante da inexperiência profissional dos recém admitidos e a necessidade de alinhamento com a rotina hospitalar, a estratégia aplicada na prática foi de reconhecer os trabalhadores mais experientes e melhor adaptados ao novo contexto. Esta situação evidenciou a sobrecarga física e mental destes profissionais na supervisão dos novos funcionários, em meio a uma dinâmica assistencial bastante tensa, devido à complexidade e gravidade dos pacientes, como visto nas falas a seguir:

*Tivemos certa dificuldade porque entraram muitos profissionais recém-formados, admitidos por processo seletivo emergencial. Estávamos no meio de uma pandemia, no caos e aí você recebe colegas novos que ainda precisam de treinamento, então essa parte foi difícil.* (E10)

*Os mais graves ficavam com os mais experientes, os menos graves para os que estavam chegando e íamos ensinando sempre. Ficávamos com 20 leitos, três enfermeiros e apenas 01 era mais experiente, eram muitos técnicos e enfermeiros novos, tivemos colegas que não conseguiram suportar essa pressão.* (E17)

A segunda subcategoria, “Exercício da liderança”, apontou como os enfermeiros fizeram para superar o medo e direcionar a equipe diante das adversidades. A necessidade de programar novos processos frente aos diversos problemas encontrados na assistência à saúde no contexto da pandemia tornou-se um ato complexo e que oportunizou novas experiências e muitos aprendizados. Por vezes, os enfermeiros sentiram limitações e insegurança, principalmente advinda de outros trabalhadores, todavia, efetivaram suas práticas de liderança, subsidiada, por vezes, pelo suporte organizacional.

Na rotina hospitalar, o dimensionamento de pessoal de enfermagem inadequado alcançou as unidades assistenciais de modo preocupante, visto que o aumento de pacientes estava ocorrendo e os trabalhadores sobrecarregados. Enfermeiros mencionaram que o déficit de profissionais fragilizava a capacidade da enfermagem de assegurar um cuidado livre de riscos. Nesse contexto, os participantes referiram que a liderança do enfermeiro foi um elemento essencial para conduzir equipes sobrecarregadas, inseguras e desmotivadas, atuando com propósito e percebendo um sentimento de apoio dos pares, em um contexto inadequado no que se refere a condições de trabalho, como notado na fala:

*A parte de liderança, os colegas não reclamaram, a gente conseguia executar todo serviço. Consegui fazer o processo de enfermagem, sabe? E o sentimento foi de medo e insegurança. Mas a liderança que o enfermeiro exerce e que eu exercia, me fazia superar esse medo.* (E16)

*Eu tive que trabalhar muito essa questão da liderança para a gente conseguir fazer o serviço fluir, porque a gente não teve apoio e era muita revolta e ao mesmo tempo os pacientes precisavam* [de nós]. *Então, a gente teve que trabalhar muito isso com a equipe, então assim, não só com a equipe de técnicos de enfermagem, também com os colegas médicos, então a gente teve até que ser líder e mostrar para eles que a gente precisava deles também.* (E11)

### Articulação multiprofissional

O treinamento e atualização profissional permitiram uma maior qualificação da assistência de enfermagem. Para esses trabalhadores, a busca constante por literatura e as práticas de treinamento deram uma maior segurança no desenvolvimento das práticas de cuidado, principalmente no início da pandemia.

O “Uso de protocolos”, subcategoria que anuncia uma estratégia descrita na dinâmica de trabalho da enfermagem e da assistência multiprofissional. Essa associação demonstrou uma maior sincronia entre variadas profissões e um fluxo assistencial mais sólido em meio à alta demanda e rotatividade de pacientes, demonstrado nas falas a seguir:

*Foram elaborados muitos POPs e protocolos, para a garantia da execução, Isso garantiu que o nosso trabalho conjunto desse certo e cada vez se aprimorasse mais.* (E5)

*A equipe multidisciplinar é bem tranquila. Não tínhamos muitos problemas. Tudo era dividido e todo mundo fazia sua parte. O fisioterapeuta, nutricionista, fonoaudiólogo, médico, era tudo bem organizado. Tudo em função do paciente, de um melhor cuidado.* (E15)

A subcategoria “Educação em saúde” corroborou para melhor aprimoramento e percepção ampla do cuidado prestado pelos integrantes da equipe de enfermagem e multiprofissional. O trabalho conjunto entre os variados profissionais possibilitou a implementação de uma rede assistencial integral, proporcionando distintas abordagens de questões específicas, como também a escolha das terapias mais adequadas aos pacientes, como percebido a seguir:

*Foi feito treinamento, foi feito também simulado né, a gente fez, então isso eu percebi que o pessoal ficou mais seguro.* (E6)

*Antes de começar a COVID-19, teve cursos, preparação, teve cursos on-line, as equipes se prepararam, fizeram protocolos.* (E14)

### Cuidado de enfermagem beira-leito

O aumento no número de pacientes graves internados, não foi acompanhado pela contratação imediata de profissionais, gerando um cenário caótico. Neste sentido, a subcategoria “Priorização dos cuidados essenciais” foi a estratégia possível visando medidas para manutenção da vida dos pacientes com COVID-19. Dentre as ações, destaca-se que como consequência, alguns registros do processo de enfermagem não foram realizados em detrimento de cuidados diretos beira leito, como citado pelos participantes.

*Aqui na emergência existe um fluxo de leitos em que fizemos a sistematização completa com histórico, evolução e prescrição. Os pacientes que se encontravam na maca a gente fazia uma evolução manual em cada turno de trabalho, a gente conseguiu manter essa parte durante a pandemia.* (E16)

*Eu tirei um plantão com três técnicos e eu de enfermeiro, três intu-bados e 25 pacientes, então, realmente um cenário de guerra.* (E2)

Não obstante, com a alta taxa de transmissibilidade do vírus, surgiram adequações nos fluxos das unidades. A reorganização das práticas assistenciais afetou a enfermagem de modo expressivo, uma vez que determinados trabalhadores não acessavam as unidades contaminadas, como o serviço de apoio do laboratório, hotelaria e nutrição, por exemplo. Desta forma, a subcategoria “Auxílio em atividades de outras áreas assistenciais” foi a estratégia que passou a ser praticada pela equipe de enfermagem, visando garantir o atendimento às necessidades humanas básicas dos pacientes. Entretanto, esta estratégia veio associada ao aumento na exposição ao vírus e a sobrecarga de trabalho, corroborando para um cuidado fragilizado e insatisfação dos trabalhadores da enfermagem, como ilustram as falas a seguir:

*A equipe de enfermagem fazia o papel de laboratório, todos os exames de sangue nós coletamos, a equipe do laboratório não podia entrar para não aumentar o fluxo de profissionais contaminantes no hospital. Para entrega de alimentos a copeira deixava na porta da emergência e o enfermeiro ia lá pegar e distribuir para cada paciente, isso era feito para poder reduzir a taxa de infecção dentro do hospital.* (E9)

*Praticamente a equipe de enfermagem desempenhava todos os papéis, transporte para exames, entregar alimentação, após avaliação da fonoaudiologia, a enfermagem ofertava a dieta assistida também.* (E2)

Assim, há que se considerar a primordialidade dos cuidados de enfermagem. Nesse tocante, notou-se a realização das práticas assistenciais focadas nas necessidades básicas, como demonstrado:

*Oferecemos um cuidado completo ao paciente. Conseguimos ter um controle melhor do que está sendo feito devido a escala de atribuição e de organização, então esse aqui vai ficar só na reanimação, esse aqui vai ficar no cuidado desses pacientes intensivos, esse vai fazer higiene, esse vai ficar só na medicação e aí eu acho que ficou um cuidado mais humanizado e sem riscos assim.* (E11)

*A gente sempre prioriza os pacientes que têm mais vulnerabilidade, a gente dá mais atenção a eles, fica mais atento, não perdendo a humanização né, porque são pacientes que não podem receber visitas.* (E9)

## DISCUSSÃO

Desenvolver estratégias para a gestão do cuidado envolvem atividades complexas e inúmeras relações no contexto laboral. Nessa perspectiva, o presente estudo evidenciou estratégias de gestão do cuidado desempenhadas no período pandêmico, mesmo diante das adversidades, nas unidades hospitalares, que contemplaram a reorganização dos serviços de saúde e fluxos e o racionamento de EPIs e insumos hospitalares, bem como a gestão de pessoas, destacando a liderança do enfermeiro e a ampliação de responsabilidades atribuídas aos trabalhadores mais experientes com a admissão emergencial de trabalhadores da enfermagem.

Uma revisão integrativa comparando o processo de trabalho de enfermeiros brasileiros com outros países de diferentes continentes apontou que ao executar o seu trabalho nas instituições de saúde que é de coordenar o processo de trabalho dos membros da equipe, orientar o processo de trabalho dos outros trabalhadores da saúde para assegurar a prestação dos serviços, estruturar o ambiente e também desempenhar procedimentos técnicos assistenciais, o enfermeiro continuamente leva em conta as necessidades dos pacientes que utilizam esses serviços de saúde^(12)^. Desse modo, os enfermeiros do presente estudo demonstraram os desafios enfrentados pela falta de insumos, o que culminou em problemas desde o cuidado direto até a gestão do cuidado em si, embora este fato não se resumisse a um caso isolado, levando em consideração todo o contexto global ^(13,14)^, como apontado também em estudos realizados nos Estados Unidos^(15)^ e China^(16)^.

Diante do cenário pandêmico, adequações diversas exigiram o uso de protocolos de cuidado e treinamentos em conjunto com a equipe multiprofissional. Mesmo com diversas mudanças, a enfermagem, ainda que sobrecarregada, continuou respondendo pela coordenação do cuidado, priorizando os cuidados diretos ao paciente e assumindo atividades de outras áreas assistenciais. O dimensionamento de pessoal de enfermagem demonstrou uma realidade comumente observada nas instituições de saúde, no entanto em relação a gestão do cuidado e exercício da liderança pelos enfermeiros, os mesmos demonstraram a efetividade do papel de líder na organização da assistência e na contratação pessoal em caráter emergencial. Corroborando com achados desta pesquisa, apesar das dificuldades, o ambiente de prática profissional do enfermeiro foi classificado como favorável antes e após a pandemia em um hospital de São Paulo ^(17)^, considerando o conjunto de intervenções e manejo dos recursos disponíveis. Nesse sentido, é válido ressaltar que a avaliação desvantajosa da adequação de equipes, bem como a discrepância de pessoal e sobrecarga de trabalho, caracteriza-se como uma problemática comum em muitos países e, apresentam-se de modo alarmante nos hospitais públicos brasileiros ^(18,19)^.

Assim como nos achados do presente estudo, as necessidades de saúde concebidas por essa pandemia ultrapassou as capacidades de hospitais e sistemas de saúde em todo o mundo, constituindo um cenário estressor para os trabalhadores da enfermagem que estão na linha de frente^(20)^. Isto porque a assistência engloba o gerenciamento contingencial de processos e recursos materiais, o que, no contexto de pandemia, foi significativo e desafiador, requerendo inclusive hospitais de campanha para dar suporte à alguns municípios no contexto nacional^(21)^. Desta forma, em cenários caóticos ou a partir do caos é possível o despertar para mudanças e a identificação de soluções ou nova organização dos elementos para manejo do problema^(22)^.

Os enfermeiros, por estarem na linha de frente, precisavam de um suporte organizacional mais evidente, tanto quanto suporte estrutural e emocional, auxiliando-os na resolução de problemas que surgiram, garantindo a segurança desses trabalhadores como também dos seus pacientes^(23)^. O dimensionamento de pessoal inadequado contribuiu para uma assistência limitada, com altas cargas de trabalho e conflitos. Portanto, estudo estruturado em evidências científicas internacionais recomenda a composição de equipes capacitadas e com número adequado de profissionais, ao mesmo passo que é apropriado limitá-los quantitativamente ao contato com indivíduos confirmados ou suspeitos, havendo equipes que atuem exclusivamente com esse público a fim de minimizar o risco de transmissão do vírus^(24)^.

Exercer a liderança nesse contexto de pandemia reitera a autonomia, a capacidade de tomada de decisão e guiados para uma prática de cuidados ética e qualificada, conforme apontam os achados do presente estudo. Consequentemente, é necessário entender a estrutura complexa da natureza humana, bem como, contemplar o meio no qual o ser humano está inserido, sendo uno com variadas características que não podem ser descontinuadas, que precisam ser consideradas nas suas relações e interrelações^(25)^. Isto posto, para além da atuação profissional em si, a ênfase em um modelo de prática voltado para as especificidades dos pacientes, suas prioridades e considerando todos os recursos disponíveis aponta um olhar sensível de reconhecimento da interdependencia das práticas asssistenciais^(7)^.

Desta forma, a atuação de enfermeiros durante a pandemia exigiu uma complexa articulação entre liderança e práticas de gestão do cuidado avançadas, para que fosse possível preservar a segurança física e minimizar os impactos emocionais da equipe de enfermagem, bem como oferecer a melhor atenção à saúde considerando as restrições nas condições de trabalho vivenciadas^(13,14,26)^.

No momento em que se considera a importância de um cuidado de enfermagem qualificado e especializado apesar das limitações em recursos materiais e humanos, é que os participantes expressaram uma rotina baseada em simulações e atualização profissional contínua nas unidades hospitalares. Há um forte consenso na literatura internacional de que as atividades de simulação são estratégias poderosas e frequentemente utilizadas para auxiliarem os profissionais de saúde a alcançarem níveis elevados de competência e cuidados mais seguros^(27,28)^. Além do mais, essas simulações e atualizações profissionais revelam se estratégias de educação permanente em saúde positiva do ponto de vista da aprendizagem significativa, com destaque para as tecnologias de educação digital^(29,30)^.

Assim, a complexidade propõe, então, uma forma de articulação dos saberes das múltiplas áreas, um novo método de ação: um método transdisciplinar, que cria, imagina, reflete, analisa as situações do cotidiano e propõe soluções^(31)^. Desse modo, a estruturação de ações bem como o envolvimento de todos os profissionais da enfermagem, como da equipe multiprofissional, é de grande assertividade para que as decisões tomadas tenham maior consentimento e possam ser implementadas com maior rapidez e agilidade, como por exemplo a criação de Comitê de Gestão de Crise multiprofissional^(32)^ e implantação de *rounds* multidisciplinares^(33)^ implantados em hospitais da região sudeste e sul do Brasil, respectivamente. Assim sendo, é imprescindível a criação conjunta de parâmetros de cuidados que sejam capazes de atingirem um alto grau de eficiência^(32,33)^.

Mesmo diante de um cenário caótico, assumindo atividades de outros profissionais, com a ausência de equipe de apoio, enfermeiros e técnicos de enfermagem lutaram por vidas e um exercício profissional digno e qualificado^(34)^. Nesse sentido, houve participação ativa nos processos de coordenação, gestão profissional e material, e de ações relacionadas a protocolos assistenciais em diferentes níveis de complexidade em outras regiões do Brasil^(35,36)^, corroborando com os achados do presente estudo.

O enfermeiro a beira leito propicia melhorias na qualidade assistencial, por meio da aplicação do processo de enfermagem com a organização do cuidado e planejamento assistencial sistematizado^(35)^. Ainda, esse modelo assistencial beira leito visa reintegrar o enfermeiro ao *locus* do cuidado assistencial direto, qualificado pela formação profissional altamente especializada^(37)^.

### Limitações do estudo

Considerando as especificidades relacionadas ao cuidado ao paciente em condição crítica, a apresentação integrada dos dados oriunda dos diferentes setores de internação pode ser entendida como limitação do estudo.

### Contribuições para a área da Enfermagem

Este estudo contribui para que a sociedade possa conhecer a atuação gerencial da enfermagem e suas repercussões na saúde da população, bem como direcionar as discussões sobre a temática nas instituições de ensino e de saúde, compartilhando as experiências de gestão. Entende-se que o potencial apresentado pela enfermagem na liderança de equipe e gestão de projetos em momentos de crise possa ser aperfeiçoado com investimentos institucionais que visem qualificar processos internos pós-pandemia.

## CONSIDERAÇÕES FINAIS

Apesar dos limites impostos pela pandemia, a atuação de enfermeiros neste período exigiu uma complexa articulação entre liderança e práticas de gestão do cuidado, as principais estratégias utilizadas estão relacionadas à reorganização dos serviços de saúde com alterações físicas e de fluxos das unidades de internação, exercício da liderança e educação em saúde.

A enfermagem pode ser caracterizada por sua atuação em complexas dimensões, entre elas, liderança e força na condução de processos gerenciais e clínicos. Deste modo, investimentos em formação de líderes, bem como a ampliação dos espaços gerenciais para enfermeiros poderão potencializar respostas para futuras crises sanitárias, qualificando o cuidado e garantindo segurança nas instituições hospitalares.
